# A machine learning based model for the precise regulation of tomato seedling growth for automatic grafting

**DOI:** 10.3389/fpls.2025.1664063

**Published:** 2025-12-11

**Authors:** Yichi Wang, Hongxuan Deng, Huiwen Li, Zeyi Mu, Song Gu, Yinghui Mu

**Affiliations:** College of Engineering, South China Agricultural University, Guangzhou, Guangdong, China

**Keywords:** plant factory, grafting seedlings, game theory, machine learning, smart light control system

## Abstract

**Introduction:**

The morphological characteristics of grafting seedlings affect the quality of automatic grafting. Because of the non-uniform and unstable lighting conditions in greenhouses, it is difficult to implement targeted control over seedlings. In contrast, plant factories are able to cultivate grafted seedlings in a more optimal environment by adjusting environmental factors like light. This research aims to propose an intelligent control method for seedling growth, in order to precisely cultivate seedlings that meet the requirements of different grafting machines.

**Methods:**

This research established an evaluation method for tomato seedlings (suitable for automatic grafting) and scored seedlings that underwent light recipe transitions at different time points. Based on the comprehensive weighting of tomato seedlings suitable for automatic grafting, combined with the growth data of seedlings under different light environments, six machine learning algorithms were used to establish growth prediction models.

**Results:**

The results indicate that the length of the hypocotyl and the diameter of the stem are crucial factors influencing whether the seedling can be mechanically grafted. And the transition of light recipes during cultivation can regulate seedling quality. XGBoost achieved the best accuracy for predicting rootstock and scion growth, with R^2^ values of 0.9253 and 0.9334, respectively. A smart light control system was established and grafting experiments were conducted. The results showed that the automatic grafting success rate and post-grafting survival rate of light- regulated seedlings were 8.3% and 1.4% higher than those of commercially available seedlings, respectively.

**Discussion:**

This demonstrates the feasibility of the model and highlights the practical application of the system in precision agriculture.

## 1 Introduction

Tomatoes stand as a crucial food crop, with widespread cultivation across the globe (Hu et al., 2016). Grafting is a common practice in tomato cultivation, employed to enhance quality and yield (Lee, 1994; Jenkins et al., 2022). It is estimated that hundreds of millions of grafted seedlings are produced each year (Lee et al., 2010; Chang et al., 2023). In order to improve the quality of the grafted seedlings and reduce the labor intensity, it is necessary to use grafting machines for the grafting process. Grafting machines have high requirements for the morphology and mechanical properties of seedlings (Kim and Hwang, 2015). Seedlings may experience impact during the automatic grafting process, which can damage them and result in slower growth (Pardo-Alonso et al., 2019). Therefore, to reduce their impact, scions and rootstocks need to have good mechanical properties. The hypocotyl needs to have a certain height and this height should be uniform (Yan et al., 2022). The stem diameters of the rootstock and the scion should be as similar as possible (Tian et al., 2013). Although different grafting machines have different requirements for the morphology and mechanical properties of grafting seedlings, the scion and rootstock must always meet the size and other specifications required by the machine (Kubota et al., 2008).

Light plays a crucial role in the growth of tomato seedlings, directly impacting their growth and physiological performance. Effective lighting can boost antioxidant activity and increase chlorophyll content in seedlings (Zhang et al., 2022). In greenhouse production, the inability to provide seedlings with suitable and uniform light results in poor uniformity, which makes it impossible to use grafting machines for grafting operations. In plant factory, using different light recipes can adjust seedling hypocotyl length and stem diameter, enhancing morphological uniformity (Zhang et al., 2024), and can also control seedling morphology within a certain range. Furthermore, seedlings can shorten their growth cycle and improve their adaptability to grafting machines through illumination, while also ensuring uniformity (Huang et al., 2025). This plays a very important role in improving the yield of grafted seedlings and promoting grafting machines. Additionally, since different grafting machines have different requirements for seedlings, suitable index weights for the automatic grafting of seedlings are proposed. These weights can effectively optimize the lighting strategy according to the requirements of different grafting machines.

Machine learning (ML) has a wide range of applications for target prediction in agricultural production. There are also many studies on the light response model of seedlings. Zhou developed a tomato seedling quality model incorporating thermal efficiency (TE) and photosynthetically active radiation (PAR) to predict the impacts of temperature and light on tomato seedling growth (Zhou et al., 2019). Niu established a inflection point model for the light response curve with the aim of photosynthesis rate, effectively improving the light energy utilization efficiency of tomato production (Niu et al., 2024). Gao has developed a photosynthesis prediction model based on machine learning to investigate the relationship between light intensity and photosynthetic efficiency (Gao et al., 2022). Singh clarified the significance and promising future of machine learning in the precision agriculture industry (Singh et al., 2025). Pero proposed precision grape cultivation techniques through machine learning and the Internet of Things, which can effectively prevent and solve grape vine diseases (Pero et al., 2024). Abuzanouneh employed machine learning and proposed an intelligent irrigation system, with the system’s maximum accuracy reaching 0.975 (Abuzanouneh et al., 2022). Machine learning also has many applications in the object recognition and classification aspects of precision agriculture. Balafas proposed a method for detecting and classifying plant diseases based on machine learning and conducted a verification (Balafas et al., 2023). Chaschatzis developed an identification model using machine learning for the purpose of detecting sweet cherry diseases. This model can make judgments about the diseases based on leaves and branches (Chaschatzis et al., 2022). Adhinata explained that through the selection of the appropriate feature combinations, machine learning can achieve high accuracy in classifying weeds and crops (Adhinata et al., 2024). Arango improved the efficiency of classifying different types of geographical areas through a series of supervised machine learning methods (Arango et al., 2016). Machine learning also has extensive applications in the precise regulation of agriculture. Chen proposed an intelligent control system, which is based on machine learning technology and can fine-tune the indoor environment according to the hydrological and meteorological processes (Chen et al., 2022). Gautam developed an offline prediction model, enabling real-time optimization control of ventilation parameters (Gautam et al., 2021). Dhal used machine learning to regulate the nutrient components of the hydroponic solution, thereby increasing the output of aquaponics (Dhal et al., 2022). Jiang established a multi-source data-driven nitrogen nutrition index optimization algorithm through RF. By adjusting the application of nitrogen fertilizer, it is possible to achieve maximum profit (Jiang et al., 2023).

However, there is a lack of predictive models for light on seedling growth, which directly regulates seedling growth.

This study explores the relationship between light and seedling morphology. It combines data-driven growth prediction models with dynamic light strategies. This approach provides precise control over seedling growth and meets the requirements for automatic grafting. This study can make developing grafting equipment easier, promote its use, encourage the integration of agricultural machinery and agronomy, and increase the economic benefits of seedling production.

## 2 Materials and methods

### 2.1 Architecture of the system

**Figure 1:**
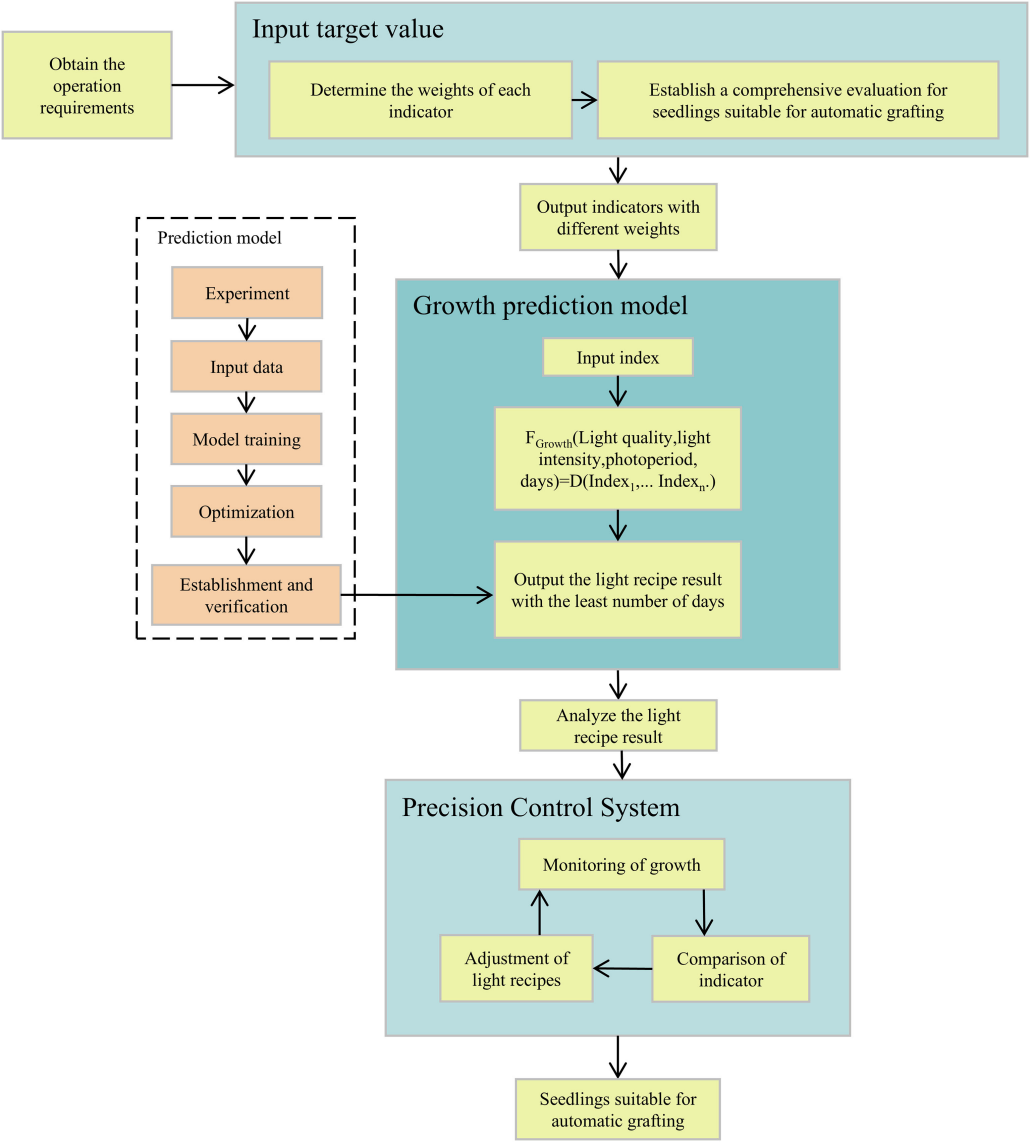
Overall architecture.

The architecture of the system is shown in **Figure 1**. The project is divided into three parts:

1. Establishing a comprehensive evaluation for seedlings suitable for automatic grafting and determining the weights of each indicator.
2. Establishing a growth prediction model.
3. Designing and verifying the smart light control system.

Details will be introduced in the following chapters.

### 2.2 Plant material

**Figure 2:**
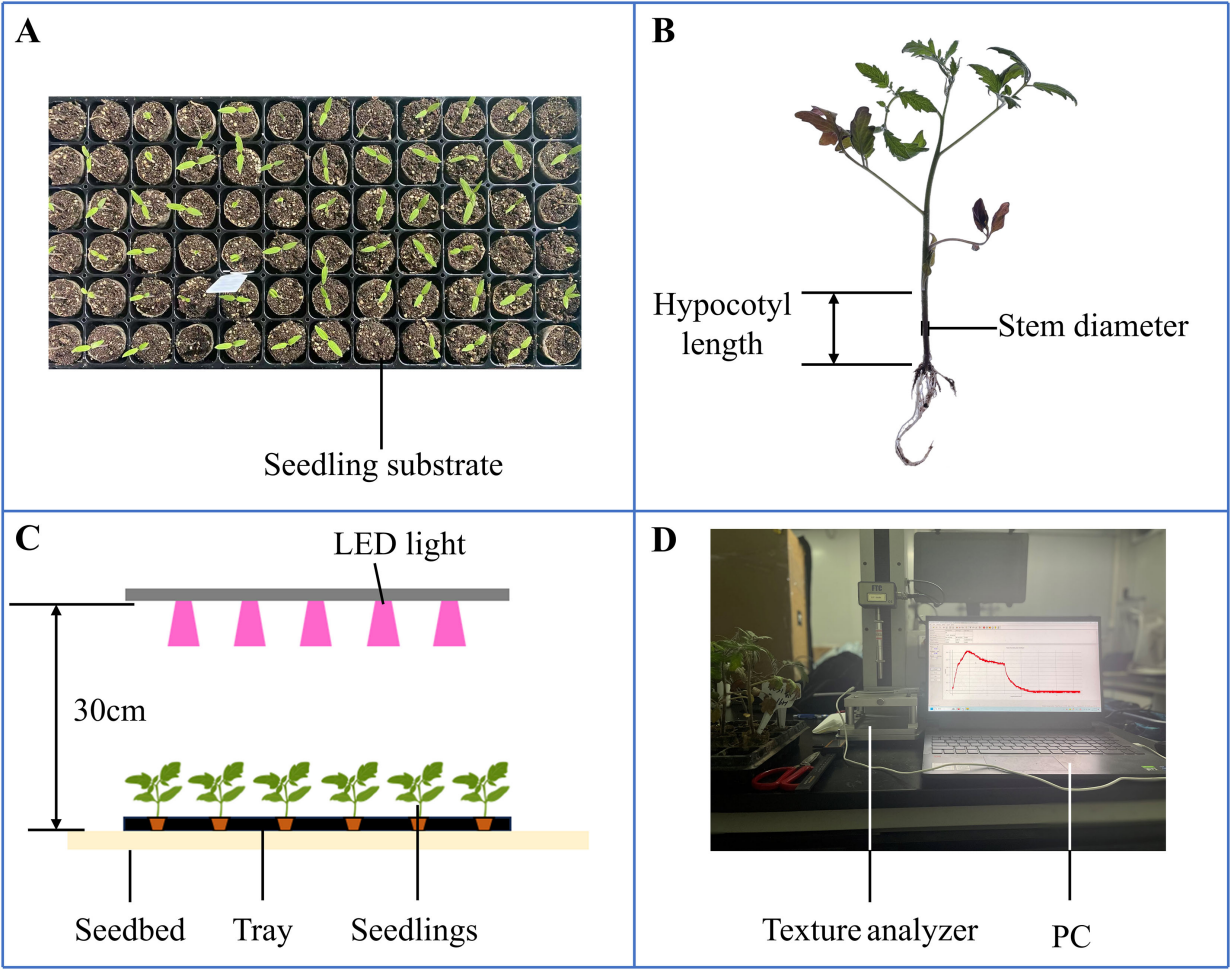
Morphology and biomechanics of tomato seeding. **(A)** Seedlings in the tray; **(B)** Morphological determination; **(C)** Light regulation; **(D)** Mechanical determination.

Both rootstock and scion seeds were obtained from Glseed Company (Guangzhou, Guangdong). The rootstock seeds (Glseed T17-2) and scion seeds (Glseed 2) were first soaked in 50°C warm water for five hours, followed by placement in an electrically heated thermostat incubator (Sunne, Shanghai) set at 26°C for germination. The rootstock seeds required an average of 3 days for germination, while the scion seeds took 4 days on average. Germinated seeds were sown into 72-hole trays (540×280×50 mm, L×W×H) filled with a substrate mixture at a ratio of grass charcoal/perlite/vermiculite = 7:2:1. And the grass charcoal we use was sourced from Pindstrup (Denmark) and its specification is 0-10mm. After the sowing process was completed, the tray needs to be placed in a water tank so that the bottom of the tray comes into contact with the water surface. This ensures that the substrate was fully soaked and that the seeds receive sufficient moisture. These trays were placed in an LED dark room within the plant factory of South China Agricultural University, where the environmental conditions were maintained at: temperature 23 ± 1°C, relative humidity 75 ± 5%, and CO_2_ concentration 580 ± 10 ppm. In experiment, each treatment of the rootstock and scion was 144 plants (2 trays), to ensure that there would be a sufficient number of uniform young plants for measurement at each sampling time point, even after possible natural losses. Throughout the experiment, tomato seedlings were irrigated with nutrient solution at intervals of two to three days. (**Figure 2**). The nutrient solution was based on the following Japanese horticultural formula (mg/L): KNO3, 808; Ca (NO3)2·4H2O, 944; MgSO4·7H2O, 492; NH4H2PO4, 153; DTPA-Fe-7, 42.9; H3BO3, 2.82; MnSO4·H2O, 1.54; CuSO4·5H2O, 0.08; ZnSO4·7H2O, 0.22; (NH4)6Mo7O24·4H2O, 0.03, respectively (Zheng et al., 2021).

#### 2.2.1 Light element experimental design

In experiments of light quality, the photoperiod was set at 12 h/d, the light intensity at 50μmol m^−2^ s^−1^, and the light quality at R: B = 100:0, 25:75, 50:50, 75:25, 0:100. In experiment of light intensity, the photoperiod was set at 12h/d, the light quality at R: B = 50:50, and the light intensity at 50,100,150,200,250 μmol m^−2^ s^−1^. In experiments of photoperiod, the light intensity was set at 200 μmol m^−2^ s^−1^, the light quality at R: B = 50:50, and the photoperiod at 10,12,14,16,18 h/d.

In experiment of light recipe transition timing, one light recipe needs to be used to promote hypocotyl elongation before changing to another light recipe to promote seedling stem increase. According to previous studies by the same authors, the hypocotyl exhibited its peak elongation rate between 5 to 9 days after emergence. A light recipe with a light quality of R: B = 3:1, light intensity of 150 μmol m^−2^ s^−1^, and a photoperiod of 18 h/d was most favorable for the elongation of the hypocotyl. A light recipe with a light quality of R: B = 1:3, light intensity of 200 μmol m^−2^ s^−1,^ and a photoperiod of 18 h/d was most conducive to increasing seedling stem thickness. Therefore, these two light recipes were selected for the light recipe transition timing experiment. The light recipe transition timings (LRTT) were to be selected on days 5, 6, 7, 8 and 9.

#### 2.2.2 Measurement methods

The rootstocks and scions were sown on 23 January 2025. The hypocotyl length and stem diameter of seedlings serve as key criteria for their suitability in automated grafting operations. On days 5, 10, 15, 20, 25, and 30 after emergence, seedlings from each treatment group were randomly sampled. Stem diameter and hypocotyl length were measured using a Vernier caliper (precision 0.02 mm), with 10 seedlings selected per sampling occasion. To avoid damage and mutual interference caused by repeated measurements on the same plant, at each sampling time point, measurements were taken on randomly selected seedlings within the treatment group In the experiment, simple random sampling was employed. The procedure involved: numbering all seedlings under each treatment, then randomly selecting 10 numbered seedlings for each measurement. Should any sampled seedlings exhibit obvious abnormalities (death, marked stunting, failure to emerge), resampling was conducted. Before and during the experiment, the vernier caliper was calibrated. For each measurement point on every seedling, the same operator used the same vernier caliper to take three replicate measurements, with the mean value recorded as the final measurement. On the 30th day, fresh and dry biomass were measured, with 10 samples selected for each determination. The same simple random sampling method was used. Seedlings were extracted from the substrate, rinsed with water, blotted to remove surface moisture, and weighed for fresh biomass using an electronic balance (precision 0.01 g). The washed seedlings were first dried at 105 °C for 30 min in an oven (Yiheng, Shanghai), then at 75 °C until reaching constant biomass. Moisture content and seedling index were calculated using the formulas described by Liu et al (Liu et al., 2022). For each mechanical parameter five biological replicates were analyzed. Mechanical characterization was performed using a texture analyzer (TMS-Pilot, FTC, Sterling, VA, USA).

### 2.3 Comprehensive evaluation of grafting seedlings based on Game Theory

The subjective weights were determined by the Analytic Hierarchy Process (AHP), following the same procedure as that in the Fuzzy AHP method, an evaluation was conducted by highly trained professionals specializing in grafting robotics to determine the criticality of various seedling parameters in relation to automated grafting processes, and the 1–9 scale method was used to construct the judgment matrix, and it needs to be verified through consistency checks. The “highly trained professionals specializing in grafting robotics” defined as “ Have over 5 years of experience in the field of robot grafting design; Gain a thorough understanding of the seedling cultivation process; Holds a bachelor’s degree or above”. The entropy method was employed to calculate the objective weights of each factor. The entropy weight method is entirely driven by the degree of data dispersion. The greater the dispersion of the data (the smaller the entropy), the higher the weight of this indicator. To prevent the composite weighting from being unduly biased towards either subjective or objective weighting, game theory is employed for the composite weighting calculation (Başeğmez, 2025). For the calculation reference of entropy weight method (He et al., 2022), for the calculation reference of AHP (Qin et al., 2024), please refer to Section 3.2 for specific evaluation.

The calculation steps of combination weight based on game theory are as follows (Yu et al., 2024) (Equation 1). The set of weight vectors *c_k_* = (*c_k1_*, *c_k2_*, …, *c_km_*) (k = 1,2 …, L) is calculated by different weighting methods, where L is the number of weighting methods and m is the number of indicators.

(1) $$c=\sum_{k=1}^{L}\alpha_{k}c_{k}^{T},c_{k}>0$$

Game theory is used to bring different weight vectors into agreement and compromise (Equation 2). The goal of minimizing the deviation of *c* and *c_k_* is achieved by optimizing the linear combination coefficient *α_k_*.

(2) $$min∥\sum_{k=1}^{L}\alpha_{k}c_{k}^{T}-c_{i}^{T}{∥}_{2}(i=1,2,\cdots ,L)$$

According to the differential properties of the matrix, the first-order derivative condition of the above formula optimization is as follows (Equation 3):

(3) $$\sum_{k=1}^{L}\alpha_{k}c_{i}c_{k}^{T}=c_{i}c_{i}^{T}$$

The linear equations corresponding to the above formula are as follows (Equation 4):

(4) $$\left[\begin{array}{cccc} c_{1}\cdot c_{1}^{T} & c_{1}\cdot c_{2}^{T} & \cdots & c_{1}\cdot c_{L}^{T}\\ c_{2}\cdot c_{1}^{T} & c_{2}\cdot c_{2}^{T} & \cdots & c_{2}\cdot c_{L}^{T}\\ \vdots & \vdots & \vdots & \vdots \\ c_{L}\cdot c_{1}^{T} & c_{L}\cdot c_{2}^{T} & \cdots & c_{L}\cdot c_{L}^{T} \end{array}][\begin{array}{c} \alpha_{1}\\ \alpha_{2}\\ \vdots \\ \alpha_{L} \end{array}]=[\begin{array}{c} c_{1}\cdot c_{1}^{T}\\ c_{2}\cdot c_{2}^{T}\\ \vdots \\ c_{L}\cdot c_{L}^{T} \end{array}\right]$$

Normalize the Linear combination coefficient (Equation 5).

(5) $$\alpha_{k}^{'}=\frac{\alpha_{k}}{\sum_{k=1}^{L}\alpha_{k}}$$

Calculate the combined weight (Equation 6).

(6) $$c^{\prime }=\sum_{k=1}^{L}\alpha_{k}^{'}c_{k}^{T}$$

### 2.4 Models for light-response prediction

**Figure 3:**
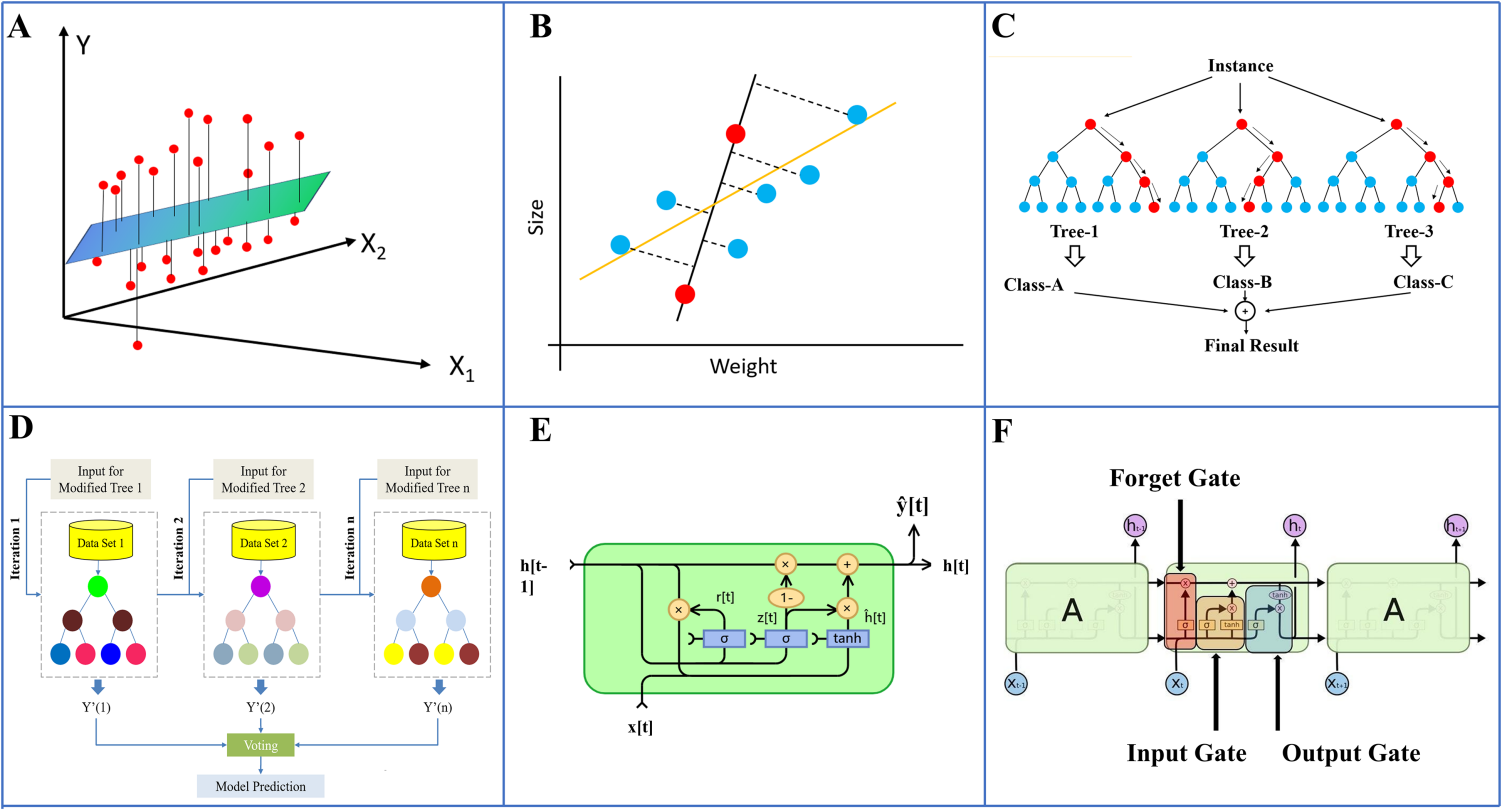
Model Architecture. **(A)** MLR, **(B)** Ridge Regression, **(C)** RF, **(D)** XGBoost, **(E)** GRU, **(F)** LSTM.

When solving multi-objective problems, six different machine learning algorithms were applied to establish the growth prediction model for tomato seedlings under various light environments. The algorithms included Multiple Linear Regression (MLR), Ridge Regression, Random Forests (RF), Extreme Gradient Boosting (XGBoost), Gated Recurrent Unit (GRU), and Long Short-Term Memory (LSTM). MLR is used to predict the linear relationship between input and output variables, assuming that the response variable follows a Gaussian distribution. The basic architecture of this model is illustrated in **Figure 3A**. Ridge Regression, a regularized variant of linear regression, employs L2 regularization to penalize large parameter values, thereby mitigating the risk of overfitting (Hoerl and Kennard, 2000), the model architecture is illustrated in **Figure 3B**. RF represents an ensemble learning approach that constructs an ensemble of decision trees and averages their predictions to enhance both prediction accuracy and generalization capability. RF is capable of capturing complex nonlinear relationships and feature interaction effects (which is precisely the typical characteristic of the interaction between environmental factors and genotypes in plant growth), and is not sensitive to outliers and assumptions about data distribution. Therefore, it can effectively model such complex relationships (Mishina et al., 2015). The model architecture is depicted in **Figure 3C**. XGBoost is an advanced gradient boosting ensemble algorithm. Its optimized gradient boosting framework minimizes the prediction error through iterative tree-based methods, supports feature importance assessment, and can handle data imbalance issues. For biological datasets with noise, XGBoost can alleviate the bias problem caused by sample imbalance through the weighted loss function (Chen and Guestrin, 2016). And the model schematic is shown in **Figure 3D**. GRU, a variant of Recurrent Neural Networks (RNNs), is designed to efficiently capture long-term dependencies in time-series data. GRU achieves the learning of long-term dependencies by updating and resetting the gates, which is in line with the biological essence of seedling growth (Chung et al., 2014). And The model architecture is illustrated in **Figure 3E**. The LSTM model, a specialized type of RNN, was proposed by Hochreiter. Similar to GRU, LSTM is an RNN with input gates, forget gates and output gates. The forget gate of LSTM can prevent the loss of early key information when modeling long sequences, but it has a higher computational complexity compared to GRU (Hochreiter and Schmidhuber, 1997). It addresses the vanishing gradient problem by leveraging memory cells and gates to capture long-term dependencies in sequential data (**Figure 3F**). In non-sequential models (MLR, Ridge, RF, XGBoost), time (number of days) is incorporated directly into the model as a feature engineering variable. This study compares non-temporal models and time-series models to determine the optimal model for predicting the growth of seedlings from the perspectives of accuracy, computational efficiency, and model complexity.

### 2.5 Deployment of the model

The system architecture was centered around the Raspberry Pi 5 platform, which supported data storage, decision execution, and seamless communication with various sensors and controllers. The platform supported multiple data formats and could process image information according to the programmed. Additionally, it connected to a range of environmental sensors to enable effective interaction between system components.

Putty software was used to establish a remote connection between the PC and Raspberry Pi for data input. When the system started running, users set the target hypocotyl length and seedling stem diameter. The Raspberry Pi then selected the lighting formula with the shortest cultivation days within the set range using a predefined inverse prediction model as the initial lighting formula. At 8 a.m. each day, the camera captured images and used OpenCV for preliminary processing, segmenting the contours and performing edge detection, and the main approach is to use the findContours and drawContours functions (Yang et al., 2022). This process yielded the seedling stem diameter and hypocotyl length. These measurement values were then transmitted to the Raspberry Pi. The system compared the actual growth parameters with the predicted parameters corresponding to the current light formula’s days. If the error exceeded 10%, the light formula required corresponding adjustments, this is dictated by the operational requirements of the grafting robot (Xie et al., 2020). The adjustment logic was as follows: if the hypocotyl length was shorter than the predicted parameter, the adjusted light formula increased the red-light ratio, reduced light intensity, and shortened the light cycle. If the hypocotyl length was longer than the predicted parameter, the opposite applied. If the seedling stem diameter was smaller than the predicted parameter, the adjusted light formula increased the blue light ratio, increased light intensity, and extended the light cycle (Hernández et al., 2016; Kim and Hwang, 2019; Kong and Zheng, 2023). If the seedling stem diameter was larger than the predicted parameter, the opposite applied. The lighting control module regulated light intensity and quality by controlling the power of the LED bulbs. An optical spectrometer (LI-180, Li-cor, USA) was used to calibrate the light intensity corresponding to the LED bulb power at a height of 30 cm above the substrate surface. Lighting calibrations performed and validated: During illumination calibration, corresponding luminous intensities were obtained by adjusting the current levels of red and blue LED bulbs. Data points were fitted to form calibration curves, generating calibration equations. During validation, a separate set of independent setpoint data is tested to ensure that, across the entire operational range, the average absolute error between the system’s set light intensity and light quality ratio and the measured light intensity and light quality ratio is less than 5%. Only then is the calibration deemed valid, as the maximum error of the optical spectrometer is 5%.The adjusted lighting formula was transmitted via a wireless module to the lighting control module for adjustment. Each time, the diameter of the seedling stem and the length of the hypocotyl were recorded, along with the corresponding light formula data. These data were added to the training set for further model optimization. After actual verification, the system’s response time to deviations is generally within 15 seconds. The system’s dynamic light formula adjustment mechanism improved the accuracy of seedling morphology and light response models, enabling precise control of seedling growth. Through the system interface, users accessed and monitored data in real-time via a PC.

### 2.6 Scoring methods

We categorized the indicators into three types: interval-target indicators, positive indicators, and negative indicators. Interval-target indicators are those for which values within a specific range or closer to a certain range are considered optimal. Positive indicators are those for which larger values are more desirable. Conversely, negative indicators are those for which smaller values are preferable. The types and ranges of indicators are shown in the **Table 1** below (Wang et al., 2024). Due to the different units and directions of each indicator, it is necessary to first standardize and unify the directions of the data, that is, convert all of them into positive indicators, and then perform weighted summation based on the weights.

**Table 1:** Types of seedling indicators.

Indicator	Type	Range	Unit
Hypocotyl length	Interval-type (target values)	[0, 150]	mm
Stem diameter	Interval-type(target ranges)	[0, 5]	mm
Moisture content	Interval-type(target ranges)	[85, 95]	%
Fresh weight	Positive	[0, 5]	g
Shoot dry weight	Positive	[0, 0.5]	g
Root dry weight	Positive	[0, 0.5]	g
Plant height	Negative	[0, 250]	mm
seedling strength index	Positive	[0, 1]	–
Radial compression force	Positive	[0, 50]	N
Axial compression force	Positive	[0, 15]	N
Bending strength	Positive	[0, 5]	MPa
Shear strength	Negative	[0, 5]	MPa

The standardized formulas are adjusted according to the direction of the indicators. Equations 7–10 respectively represent the standardized formulas for positive indicators, negative indicators, interval-type indicators (target values), and interval-type indicators (target ranges).

(7) $$T=\frac{v-L_{min}}{L_{max}-v}$$

(8) $$T=\frac{L_{max}-v}{L_{max}-L_{min}}$$

(9) $$T=1-\frac{\left|v-a\right|}{\text{max}(a-L_{min},L_{max}-a)}$$

(10) $$T(a)=\{\begin{array}{cc} 1 & \text{if}a\;\in \;[s_{min},s_{max}]\\ \frac{a-L_{min}}{s_{min}-L_{min}} & \text{if}a\;\in \;[L_{min},s_{min})\\ \frac{L_{max}-a}{L_{max}-s_{max}} & \text{if}a\;\in \;(s_{max},L_{max}]\\ 0 & \text{if}a\;<L_{min}\text{or}a>L_{max} \end{array}$$

In the formula, *T* means the standardized value, *v* means the actual value, $L_{min}$ means the minimum value of the range, $L_{max}$ means the maximum value of the range, *a* means the target values, $s_{min}$ means the minimum value of the target ranges, $s_{max}$ means the maximum value of the target ranges. For the purposes of this study, taking the operational requirements of the grafting robot as an example, When calculating the score for the diameter of the seedling stems, $s_{max}$ is set to 3.3mm and $s_{min}$ to 2.7mm. When calculating the moisture content score of the seedlings, $s_{min}$ and $s_{max}$ are respectively the same as $L_{min}$ and $L_{max}$.

The standardized values that have been calculated are used to calculate the comprehensive score according to Equation 11, and the maximum score is 1 point.

(11) Comprehensive score=∑ (standardized value×comprehensive weight)

### 2.7 Implementation details

All data analysis, statistical modeling, and predictions were implemented in Python (v3.8.12) using the libraries summarized in **Table 2**. The key parameters for the machine learning models are also listed in the table.

**Table 2:** Software libraries and key parameters used for machine learning modeling.

Category	Tool/library name	Version	Key parameters/notes	Citation
Programming Language	Python	3.8		(Coelho and Richert, 2015)
Data Processing	pandas	1.3.3		(Muntin et al., 2023)
	NumPy	1.21.2		
Machine Learning (Classical)	scikit-learn	1.0.2	MLR: Default parameters.	
			Ridge Regression: alpha=1.0	(Pedregosa et al., 2011; Shaik et al., 2022)
			RF:n_estimators=100,random_state=42	
Gradient Boosting	XGBoost	1.5.0	n_estimators’: 200, learning_rate’: 0.1, max_depth: 6, subsample’: 0.8, random_state’: 42	(Agrawal et al., 2022)
Deep Learning	TensorFlow & Keras	2.8.0	GRU:optimizer=‘adam’,loss=‘mean_squared_error’, epochs=200, units=64	(Park et al., 2022; Liu et al., 2023)
			LSTM:optimizer=‘adam’,loss=‘mean_squared_error’, epochs=200, units=64	

## 3 Results

### 3.1 Light element experiment results

**Figure 4:**
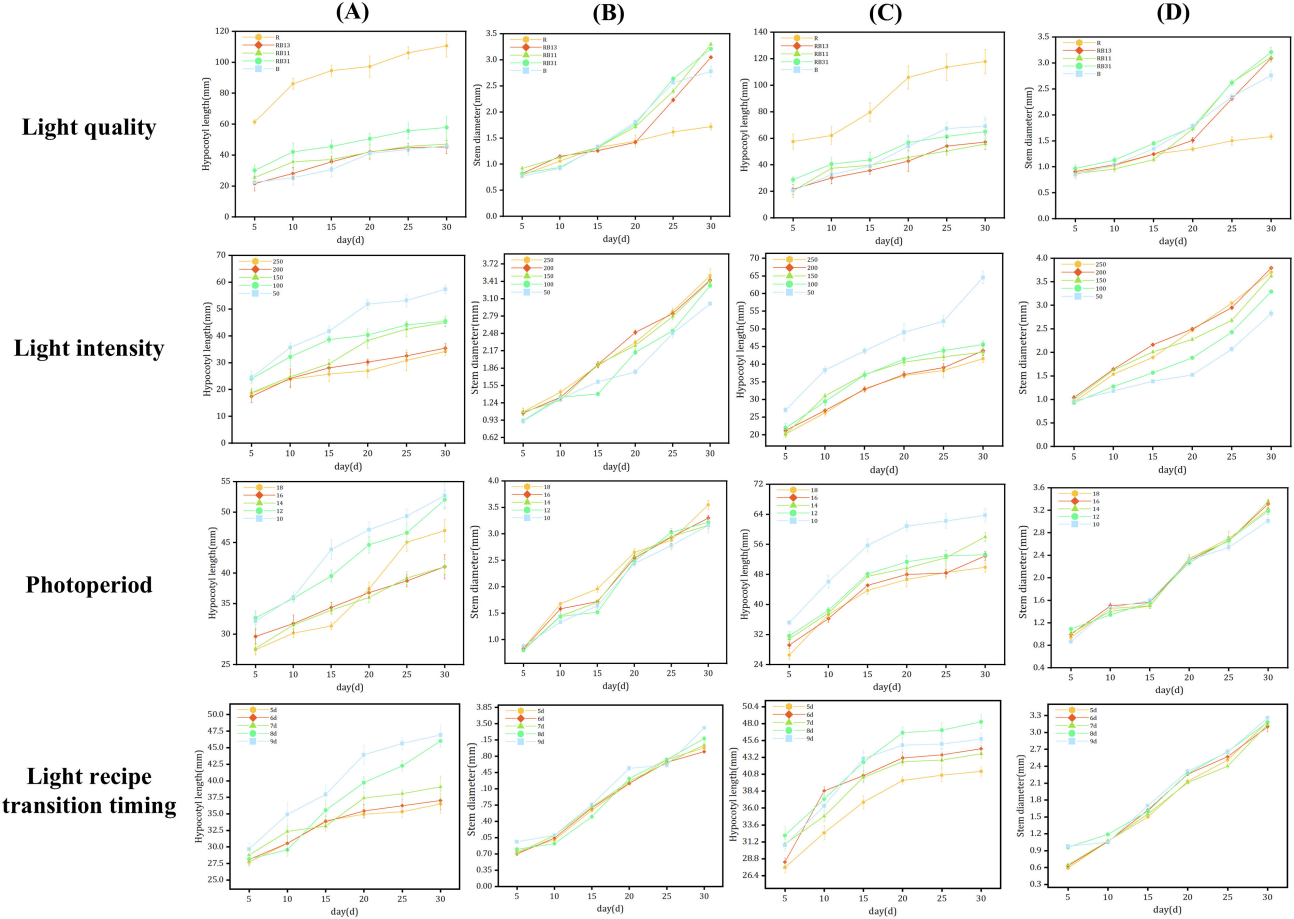
Data on light quality, light intensity, photoperiod, and LRTT experiments. **(A)** Length of the hypocotyl of rootstocks, **(B)** Diameter of the stem of rootstocks, **(C)** Length of the hypocotyl of scions, **(D)** Diameter of the stem of scions.

The total sample size is 2808 plants, and light quality, light intensity, photoperiod and LRTT had different effects on seedling growth (**Figure 4**). Increasing light intensity was found to effectively suppress hypocotyl elongation while promoting stem diameter growth. Conversely, shorter light durations were observed to facilitate hypocotyl elongation but inhibit stem development in seedlings. Different LRTTs (light recipe transition timing) affect seedling morphology. Within a certain range, the later the timing of light recipe transition, the longer the length of the hypocotyl of seedlings. The optimal LRTT for both rootstock and scion is 8^th^ day, this is determined based on the score (**Table 3**).

**Table 3:** Scores of grafting seedlings with different LRTT.

Type	LRTT 5^th^	LRTT 6^th^	LRTT 7^th^	LRTT 8^th^	LRTT 9^th^
Scion	0.729	0.717	0.728	**0.766**	0.742
Rootstock	0.533	0.562	0.557	**0.662**	0.631

Bold indicates the value with the highest score in the table.

### 3.2 Comprehensive evaluation of tomato seedlings suitable for automatic grafting

**Figure 5:**
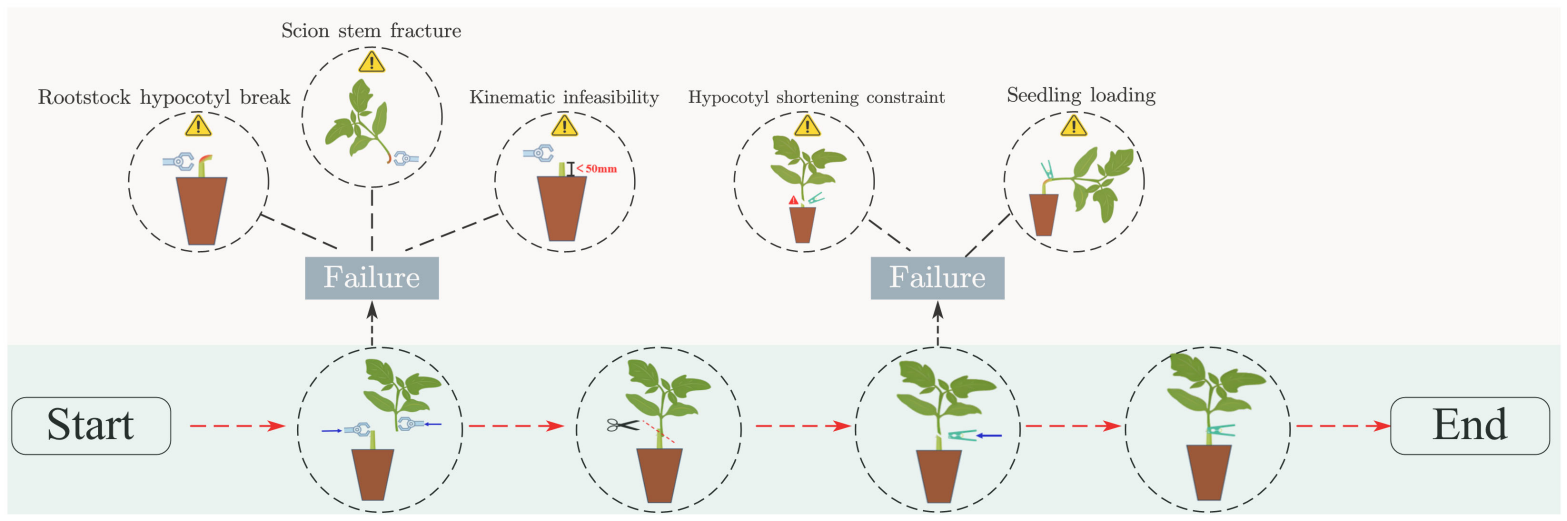
Automated mechanical grafting process.

As illustrated by the grafting machine designed by Xie (Xie et al., 2020), the rootstock and scion undergo distinct clamping and cutting processes, respectively. First, the crown-removed rootstock and the upper part of the cut scion are brought into parallel contact through a mechanical structure. Then, a knife is used to make a slanted cut at the contact point, and the two parts are connected with a grafting clamp to form a grafted seedling. The grafted seedlings are returned to their designated positions through a transport module. The rootstock should have as large as possible radial compressive strength, axial compressive strength, bending strength, and as small as possible shear strength. The scion should have as large as possible radial compressive strength, bending strength, and as small as possible shear strength. The axial compression force of the scion has little effect on the success rate of automatic grafting. Except for plant height, other characteristics such as fresh biomass are better when higher. If the hypocotyl length and stem diameter of seedlings do not meet the requirements of the grafting machine, the grafting operation will fail directly (Wang et al., 2024) (**Figure 5**). Excessive deviation in hypocotyl length will cause significant vertical misalignment in the clamping position of the grafting clip, resulting in unstable connection of grafted seedlings and subsequent scion detachment, thereby directly compromising grafting success rate. Similarly, excessive deviation in stem diameter prevents tight contact between the cut surfaces of rootstock and scion after grafting, leading to infection-induced mortality and ultimately reducing post-grafting survival rate. According to practical requirements, the grafting robot is designed with an operational tolerance of 10%. This means that seedlings with hypocotyl and stem diameters varying within ±10% of the target value can undergo robotic grafting operations stably (**Figures 4**, **5**).

**Figure 6:**
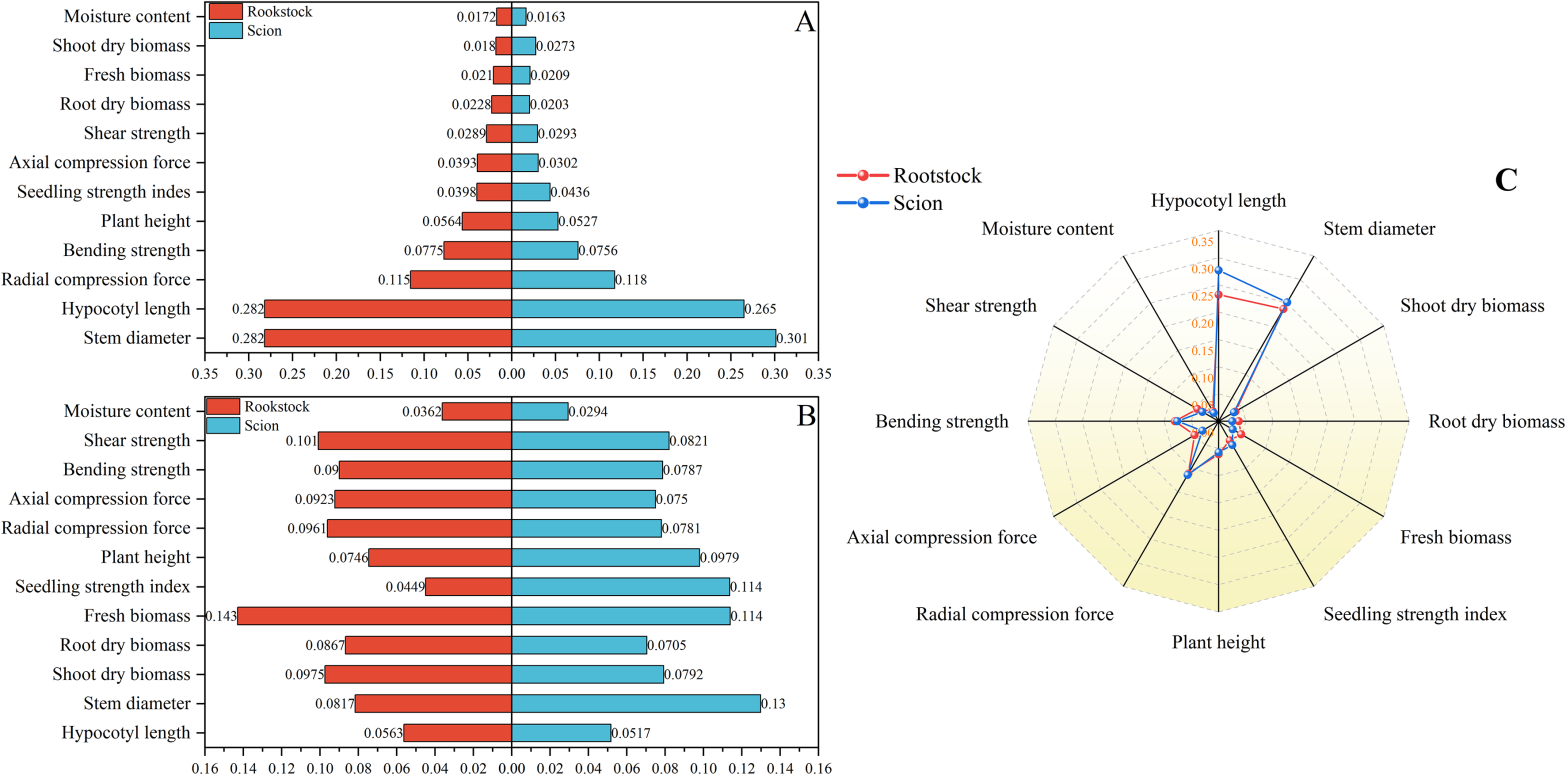
Weights of rootstocks and scions suitable for automatic grafting. **(A)** Subjective weights obtained by AHP. **(B)** Objective weights obtained by entropy method. **(C)** Comprehensive weights based on game theory. The numbers represent the corresponding weights of each indicator.

From the morphological indicators (C1) and mechanical properties (C2), a total of 12 indicators were selected to evaluate tomato seedlings suitable for automatic grafting, including hypocotyl length (C1-1), seedling diameter (C1-2), shoot dry biomass (C1-3), root dry biomass (C1-4), fresh weight (C1-5), plant height (C1-6), seedling strength index (C1-7), moisture content (C1-8), radial compression force (C2-1), axial compression force (C2-2), bending strength (C2-3), and shear strength (C2-4). According to the process of automatic grafting of seedlings, different subjective weights are assigned to the rootstock and scion respectively. After combining the objective weights, the resulting comprehensive weight is obtained. The weights of the length of the hypocotyl and the diameter of the seedling stem are much higher than those of other indicators (**Figure 6**). Therefore, how to make the length of the hypocotyl and the diameter of the seedling stem meet the requirements of the grafting robot is the key to cultivating suitable seedlings for automatic grafting. The operational mechanisms of disparate grafting apparatuses vary, thus giving rise to distinct prerequisites for seedling morphology. Consequently, for different grafting machines, the comprehensive weight of seedlings may exhibit slight variations.

Based on comprehensive weighting, seedlings undergoing light recipe transitions at different timing were comprehensively evaluated, the optimal length of the hypocotyl was set at 50mm, and the optimal diameter range of the seedling stem was 3 - 3.5mm (Xie et al., 2020) (**Table 3**). Under the same growth conditions, both the rootstock and scion of LRTT 8^th^ achieved the highest scores, with significant differences in scores among different LRTT. LRTT has a peak, the score of LRTT 9^th^ does not improve compared to LRTT 8^th^. Overall, using multiple light recipes during the seedling growth cycle can effectively change seedling morphology and is one of the methods for precisely regulating growth.

### 3.2 Modeling

#### 3.2.1 Model select

**Figure 7:**
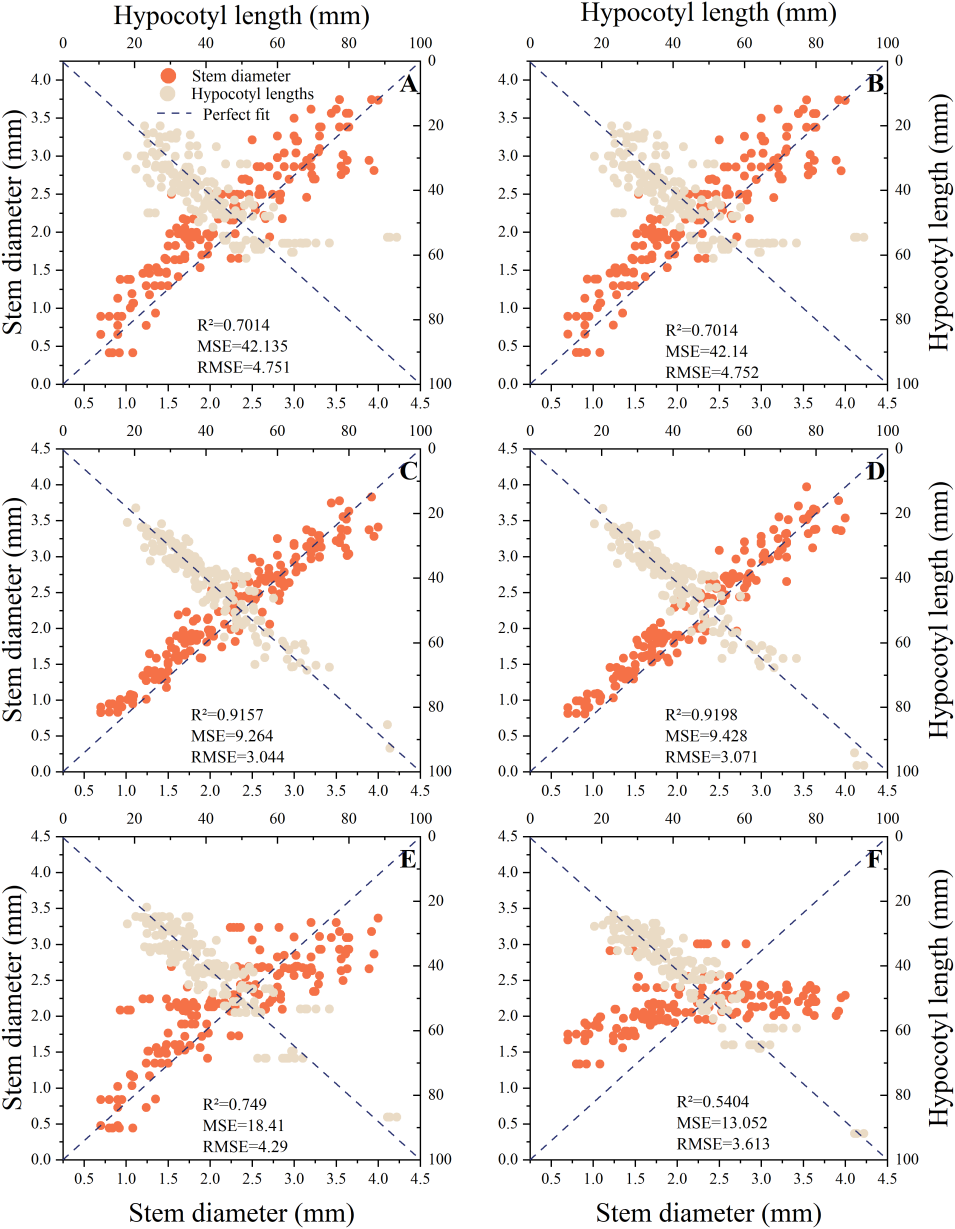
Fitting results of different prediction models for rootstocks. **(A)** MLR, **(B)** Ridge Regression, **(C)** RF, **(D)** XGBoost, **(E)** GRU, **(F)** LSTM.

**Figure 8:**
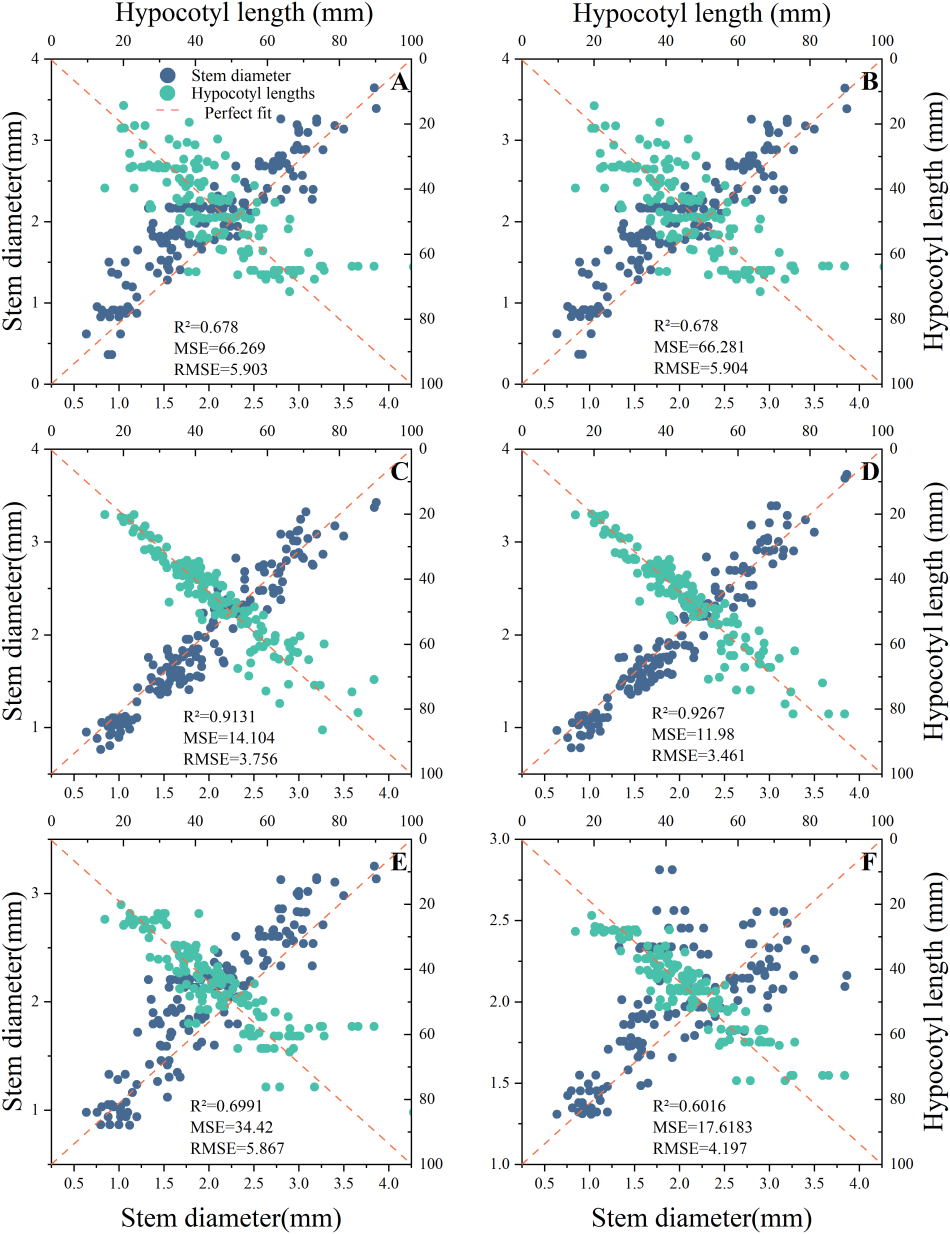
Fitting results of different prediction models for scions. **(A)** MLR, **(B)** Ridge Regression, **(C)** RF, **(D)** XGBoost, **(E)** GRU, **(F)** LSTM.

Based on the comprehensive weights in section 3.1, it can be concluded that the hypocotyl length and stem diameter of seedlings have the greatest impact on automatic grafting. Therefore, this study uses hypocotyl length and stem diameter as indicators. This study organized the hypocotyl length and stem diameter of seedlings under different light environments according to corresponding time and light recipes into a database. The database contains 849 data entries for rootstocks and 763 data entries for scions. This study used time-series partitioning’ approach to selected all data collected from the first 80% of time points as the training set, while data from the subsequent 20% of time points served as the test set. Six machine learning models were used to input time and light recipes and obtain prediction models for the length of the hypocotyl and the diameter of the seedling stem. **Figures 7**, **8** show the key performance indicators of rootstocks and scions using these models, respectively. Combined with the comprehensive weight, the comprehensive R ² of the model takes the weighted average value of the output R ².”Perfect fit” refers to a situation where the predicted value and the actual value are exactly the same (with an error of 0), and at this point, R² equals 1. The closer the points cluster around this diagonal line, the better the model’s predictive performance.

The findings indicated that there were substantial discrepancies in the efficacy of employing various models to predict the hypocotyl length and stem diameter of seedlings. The predictive accuracy of all models diminishes as the length of the scion hypocotyl increases, indicating the presence of systematic bias. A probable cause lies in the limited number of samples with long hypocotyls within the scion dataset. During model training, this reduced sample size diminished optimization for this segment, resulting in inadequate fitting of extreme values and consequently generating bias. Therefore, the current model is most reliable for predicting the rootstock hypocotyl length of the grafts that are 80mm or less in length.

Additionally, these models exhibited divergent impacts on the prediction of rootstock and scion morphology. According to R ², the three best fitting models for rootstock prediction are XGBoost (0.9198), RF (0.9157), and MLR (0.9105), and XGBoost and RF have smaller MSE and RMSE values. The three best fitting models for scion prediction are XGBoost (0.9267), RF (0.9131), and MLR (0.8981). XGBoost has the smallest MSE and RMSE values, only 11.98 and 3.461, respectively. Compared with MRL, XGBoost reduced MSE by approximately 81.92% and RMSE by approximately 57.49%. According to Perfect Fit, the accuracy of MLR and Ridge Regression in predicting the hypocotyl length of rootstocks gradually decreases with increasing length. In the context of predicting the stem diameter of rootstock seedlings, the LSTM model exhibited a pronounced tendency towards bias, in contrast to the XGBoost and RF models, which demonstrated a comparatively lesser degree of prediction bias. In the context of predicting scion hypocotyl length, the MLR model exhibited a higher degree of deviation compared to other models. It is noteworthy that the prediction accuracy of all models exhibited a decline in accuracy with increasing scion hypocotyl length. For the prediction of scion seedling stem diameter, the prediction accuracy of LSTM is the worst. A comparative analysis of XGBoost and RF reveals that both models exhibit commendable stability and accuracy in their predictive capabilities. However, XGBoost demonstrates slightly superior prediction and training times compared to RF, at 0.1488 seconds and 0.1292 seconds, respectively (**Table 4**). Consequently, XGBoost is identified as the better predictive model.

**Table 4:** The performance of different prediction models for seedlings.

Type	Model	Training time (s)	Prediction time (s)
Rootstock	MLR	0.0021	0
	Ridge Regression	0.0031	0.001
	RF	0.3486	0.027
	XGBoost	0.1488	0.002
	GRU	0.1013	0.0568
	LSTM	0.0952	0.0587
Scion	MLR	0.002	0
	Ridge Regression	0.0031	0.001
	RF	0.3243	0.027
	XGBoost	0.1292	0.002
	GRU	0.0988	0.0579
	LSTM	0.0951	0.051

#### 3.2.2 Optimization of hyperparameters in model

**Figure 9:**
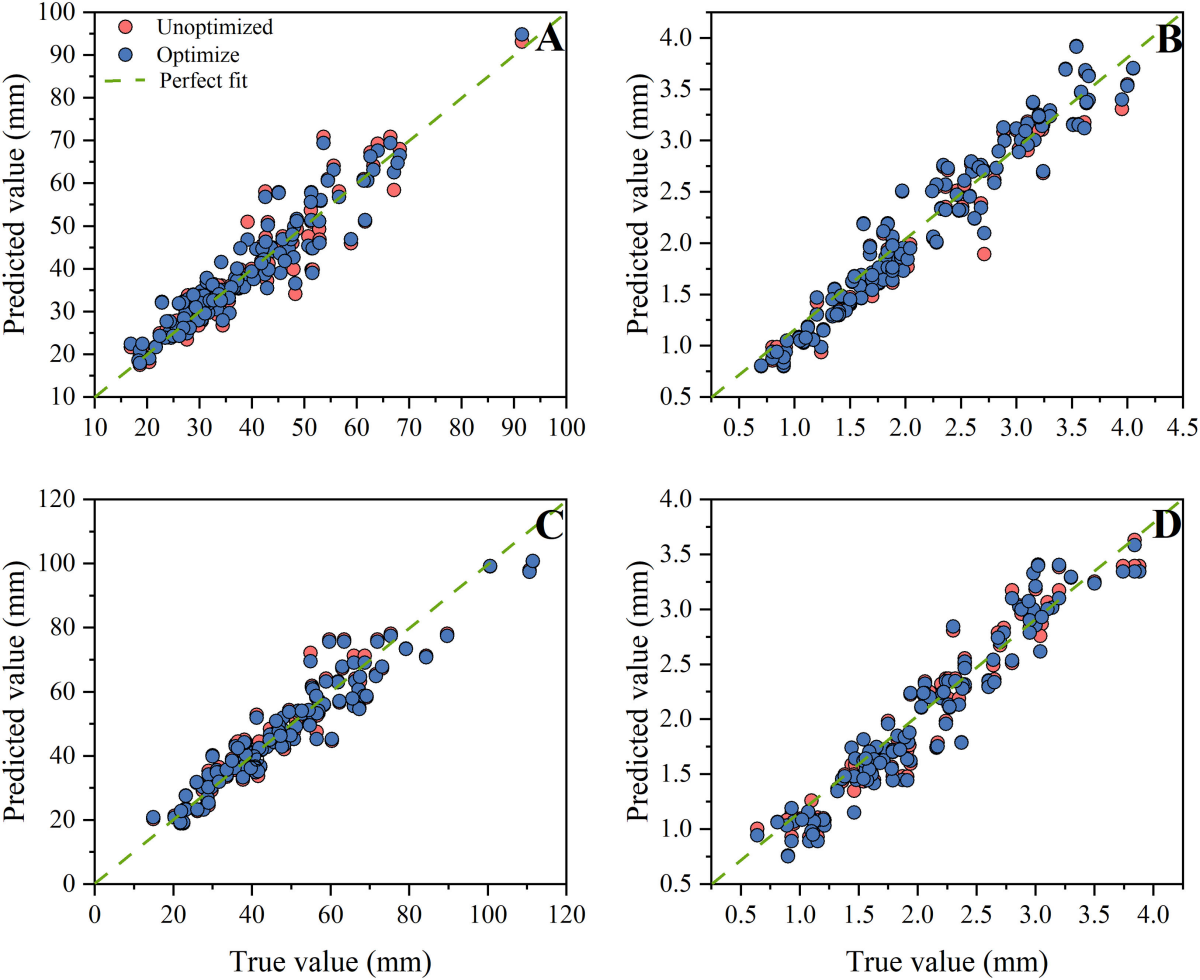
Comparison between model optimization before and after. **(A)** Rootstock hypocotyl, **(B)** Rootstock stem, **(C)** Scion hypocotyl, **(D)** Scion stem.

In order to enhance the prediction accuracy of the model, it is necessary to optimize XGBoost model. After checking the data quality, add Gaussian noise (noise level 0.01) and feature value perturbation (perturbation factor 0.05) to enhance the data, to improve model robustness (Wang et al., 2022; Zhou et al., 2024). The selection of numerical values is based on the reference literature (Yu et al., 2022). Data augmentation was only applied on the training set, while the test set remains in its original, unenhanced state for the final evaluation of the model’s generalization performance. The application of Bayesian Optimization facilitates the identification of the optimal combination of parameters, including the learning rate and subsample, while Early Stopping is employed for the estimation of relevant parameters. The Bayesian optimizer dynamically models the parameter-performance relationship using a Gaussian process surrogate model and efficiently approximates the optimal solution through 50 iterations. All random processes are fixed with a seed of random_state=42 to ensure reproducibility of the experiments, and parallel computing (n_jobs=-1) significantly improves the search efficiency. For each set of hyperparameters to be evaluated, the model training will automatically terminate after early_stopping_rounds=50 consecutive rounds without an improvement in the performance on the validation set (in this study, the negative mean squared error). The optimization process aims to minimize the mean square error of the validation set, and the performance of each group of parameters is evaluated using 5-fold cross-validation. A comparison of the results before and after optimization reveals that the optimized XGBoost model exhibits enhanced accuracy in predicting the length of the hypocotyl and the diameter of the seedling stem (**Figure 9**). The performance comparison of the optimized model is shown in **Table 5**. The R² value of the model for predicting the length of the hypocotyl of rootstocks improved from 0.8979 to 0.9048, an improvement of 0.76%, while the MAE and MSE improved by 5.08% and 15.6%, respectively. The R² value of the prediction model for the stem diameter of rootstocks was improved from 0.9417 to 0.9457, representing an improvement of 0.43%. The MAE and MSE were improved by 2.61% and 6.85%, respectively. The R² value of the prediction model for the length of the hypocotyl of the scion exhibited an enhancement of 0.87%, while the MSE demonstrated an improvement of 8.62%. The R² value of the prediction model for the stem diameter of the scion exhibited an enhancement of 0.56%, while the MSE demonstrated a reduction of 12.7%. The rootstock prediction model demonstrated a comprehensive R² of 0.9253, while the scion prediction model exhibited a comprehensive R² of 0.9334. In summary, the optimized positive prediction model demonstrates a higher degree of accuracy.

**Table 5:** Performance comparison before and after model optimization.

Indices	Unoptimized				Optimized			
	HL(R)	SD(R)	HL(S)	SD(S)	HL(R)	SD(R)	HL(S)	SD(S)
MAE	3.4375	0.154	4.402	0.159	3.2628	0.1503	4.158	0.142
MSE	24.5805	0.044	33.582	0.041	20.7465	0.0413	30.918	0.035
RMSE	4.9579	0.211	5.795	0.201	4.5548	0.2032	5.56	0.187
R²	0.8979	0.9417	0.9224	0.931	0.9048	0.9457	0.9305	0.9363

R is Rootstock, S is Scion, HL is Hypocotyl Length, SD is Stem Diameter.

#### 3.2.3 Establishment of reverse prediction model

**Figure 10:**
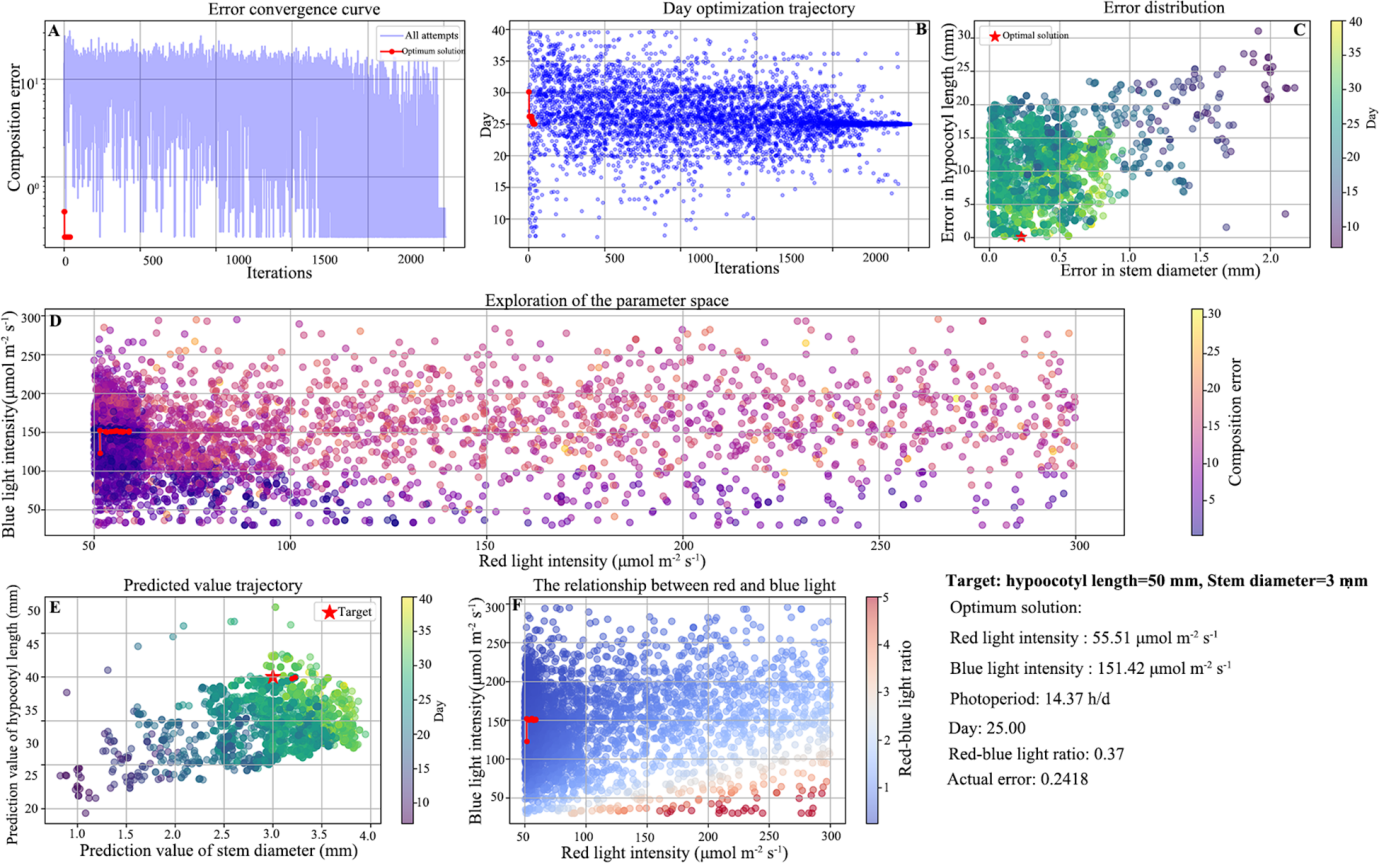
Optimization routine. **(A)** Error convergence curve, **(B)** Day optimization trajectory, **(C)** Error distribution, **(D)** Exploration of the parameter space, **(E)** Predicted value trajectory, **(F)** The relationship between red and blue light.

Establish a reverse prediction model based on the established positive prediction model by setting the range of light recipes and days. In order to get the solution with the minimum error and the minimum number of days, the differential evolution (DE) was used for optimization, and the red-blue light ratio was set as a constraint. The two-stage optimization method was employed. In the first stage, the light recipe solution that met the accuracy requirements was found. In the second stage, among the solutions that met the requirements, the one with the shortest number of days was identified. This can be achieved by finding the solution with the shortest growth period among all the light recipes that meet the prediction accuracy requirements. To achieve a balance between the search capability and result reliability of the parameter coordination algorithm, the configuration of DE is as follows, based on the referenced literature (Cao et al., 2012; Kaur et al., 2019; Gutiérrez et al., 2022): The ‘best1bin’ mutation strategy was used, which combines the information of the current population’s best individual with a random differential vector to balance global exploration and local exploitation. The population size was set to 15, and the scaling factor F is dynamically and adaptively sampled within the interval [0.5, 1), with the crossover probability CR fixed at 0.7. The optimization process terminates when the maximum number of iterations (maxiter = 1000) was reached or the improvement of the best solution between consecutive generations is lower than the tolerance (tol = 0.01). To improve the solution accuracy, the local polishing option (polish = True) was enabled at the end. All random processes were controlled by a fixed seed (seed = 42) to ensure complete reproducibility of the experiment. The error represents the Euclidean distance between the calculated predicted value and the target value, to ensure that the global optimum solution is not missed during the first phase, set the error threshold to less than 0.3. Set the red-light intensity and blue-light intensity ranges from 50 to 300 μmol m^−2^ s^−1^, the photoperiod ranges from 10 to 18 hours per day, and the number of days from 3 to 40 days. For the grafting machine designed by Xie, the suitable seedling size is preset as follows: the hypocotyl length of 50 mm and the seedling stem diameter of 3 mm. The optimization process is shown in **Figure 10**. It can be seen that the accuracy of the reverse prediction model for the length of the hypocotyl and the diameter of the seedling stem gradually increases with the number of days. The optimal solution obtained: red light intensity was 55.51 μmol m^−2^ s^−1^, blue light intensity was 151.42 μmol m^−2^ s^−1^, photoperiod was 14.37 h/d, red- blue light ratio was 0.37, error was 0.2418, and predicted growth time was 25 days.

### 3.3 Model effect evaluation based on system implementation

**Figure 11:**
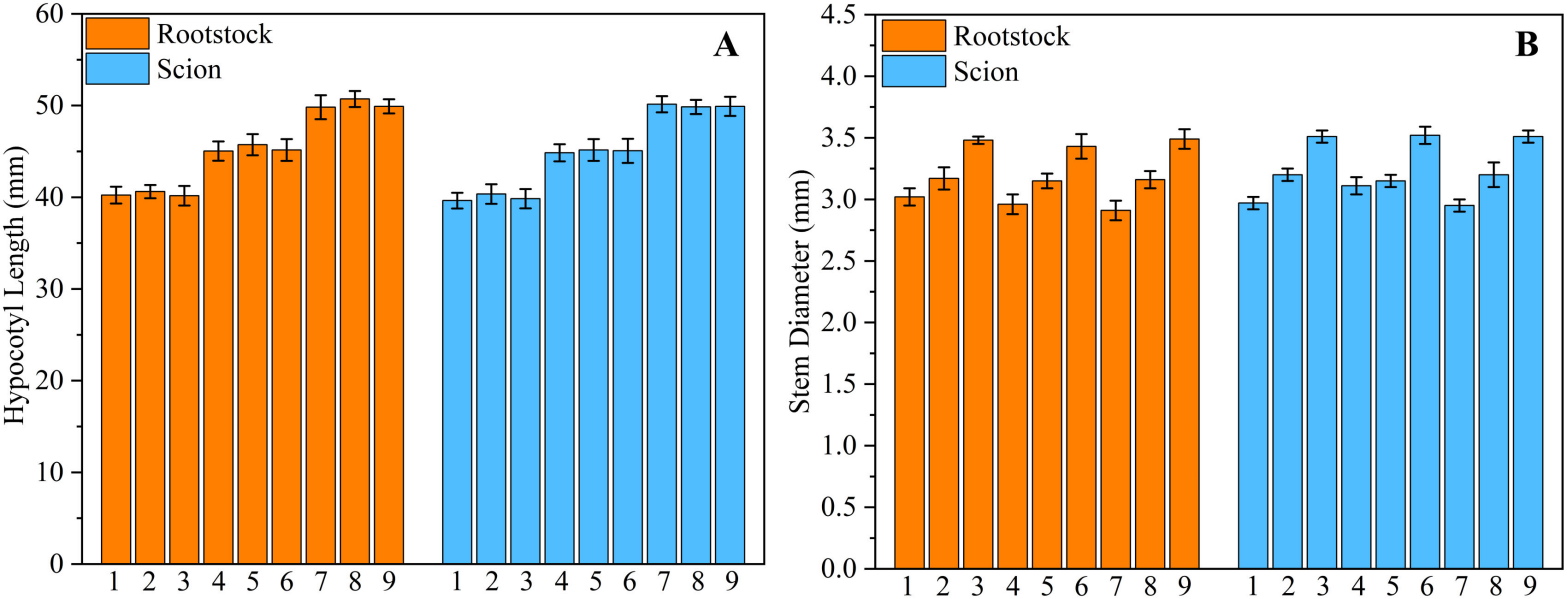
Intelligent light control system validation experiment. **(A)** Hypocotyl Length, **(B)** Stem Diameter.

To verify that the model can precisely cultivate seedlings with different requirements, a light control system validation experiment was designed. The experiment was conducted separately for rootstocks and scions, with preset target hypocotyl lengths of 40, 45, and 50 mm and target stem diameters of 3, 3.2, and 3.5 mm. A two-factor three-level full factorial design was adopted, resulting in a total of nine experimental groups (**Table 6**). In experiment, each treatment of the rootstock and scion was 144 plants (2 trays). For each experimental group, 20 rootstocks and scions were randomly selected for measurement. The measurement results were the actual values. A paired t-test was conducted using SPSS 19.0 ((lBM, Inc., Chicago, IL, USA) for each actual value and its corresponding predicted value. The purpose of this test was to prove whether there was a significant deviation between the cultivated seedlings by the system and the actual values **Figure 11**.

**Table 6:** Two-factor three-level experimental.

Experiment number	Target length of the hypocotyl(mm)	Target diameter of the stem(mm)
1	40	3
2	40	3.2
3	40	3.5
4	45	3
5	45	3.2
6	45	3.5
7	50	3
8	50	3.2
9	50	3.5

The results show that the maximum deviation between the actual hypocotyl of the light-regulated seedlings and the predicted value did not exceed 3%, and the maximum deviation between the actual stem diameter of the seedlings and the predicted value also did not exceed 3%. The P-values of all paired t-tests were all greater than 0.05, which indicates the effectiveness of the light control system.

**Figure 12:**
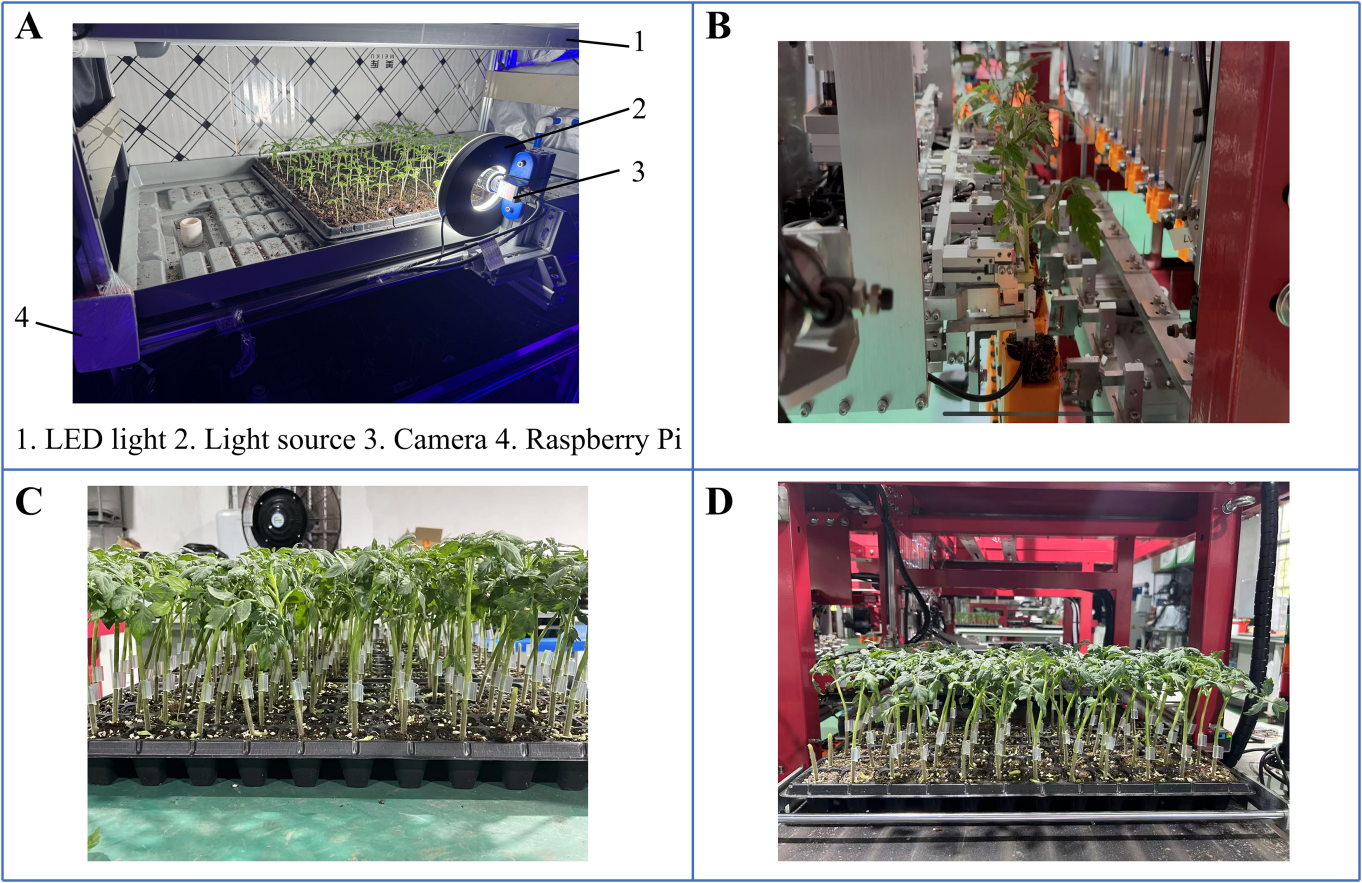
Model validation grafting experiment. **(A)** Intelligent light control system, **(B)** Automatic grafting experiment, **(C)** Light- regulated seedlings after grafting, **(D)** Commercially available seedlings after grafting.

**Figure 13:**
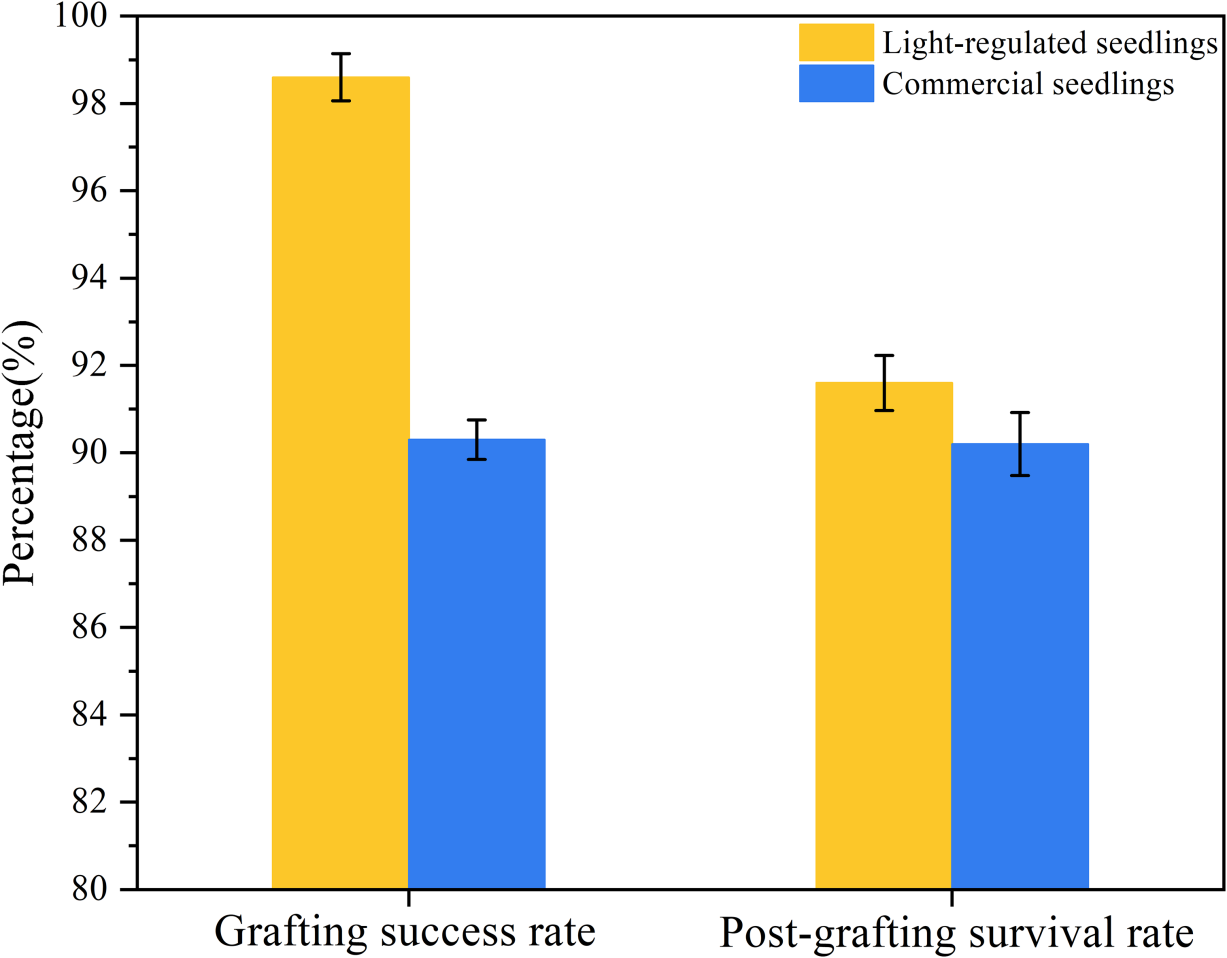
Results of automatic grafting experiments.

Model validation grafting experiment was conducted to compare seedlings cultivated using an intelligent light control system with commercially available seedlings (**Figure 11A**). The experiments were carried out in the Intelligent Equipment Research Laboratory for Protected Horticulture at South China Agricultural University. The automatic grafting machine used in the experiments was designed and manufactured by Xie (**Figure 12B**). The commercially available seedlings were purchased from Glseed Seed Farm in Sanshui City, Guangdong Province (112°54’E,23°17’N), the planting time was in April, and are of the same variety as the light-regulated seedlings. The selected commercially available seedlings are all within the grafting period, consistent with the light-controlled seedlings. The grafting procedure was carried out by researchers unaware of the treatment to minimize subjective bias. A total of 10 trays of commercially available seedlings were included, consisting of 5 trays of rootstocks and 5 trays of scions. Additionally, 10 trays of light-regulated seedlings were used, also comprising 5 trays of rootstocks and 5 trays of scions. All trays employed had a 72-hole configuration. Compare the success rate of automatic grafting operations between seedlings cultivated under two different conditions, as well as the survival rate after grafting is completed. The grafting results are shown in **Figures 12C, D**. The results indicate that the automatic grafting success rate of the light-regulated seedlings was 98.61%, which was 8.3% higher than that of the commercially available seedlings. The survival rate after grafting was 91.67%, which was 1.4% higher than that of the commercially available seedlings (**Figure 13**).

This predictive model can accurately cultivate seedlings suitable for automatic grafting according to demand, and the automatic grafting success rate and healing rate of the seedlings are superior to those of commercially available seedlings. This is because the morphological uniformity of light-regulated seedlings is better, and more seedlings meet the requirements of the grafting machine in each tray.

## 4 Discussion

### 4.1 Patterns of light effects on seedling growth

The regulation of light on the growth of tomato seedlings had certain regularity. The three elements of light (light quality, light intensity and photoperiod) had different effects on seedling growth (Hernández et al., 2016; Jin et al., 2023). An increase in the proportion of red light promoted the elongation of the hypocotyl, while an increase in the proportion of blue light promoted the enlargement of the seedling stem (Yousef et al., 2021). However, monochromatic blue light caused the hypocotyl of seedlings to elongate (Kong and Zheng, 2023), and monochromatic light was less effective than combined light for seedling cultivation (Liu et al., 2018). The intensity of light significantly affected the accumulation of dry matter in tomato seedlings (Park et al., 2020; Zheng et al., 2023). Higher light intensity inhibited the length of the hypocotyl and increases the stem length of the seedlings, while lower light intensity causes the hypocotyl of tomato seedlings to elongate (An et al., 2021). Within a certain range of light intensity, the higher the light intensity, the faster the seedlings grow. However, light intensity exceeding this range would inhibit seedling growth. This was because seedlings exhibit light inhibition (Huber et al., 2021; Chen et al., 2022). There was also a threshold for photoperiod. Once the threshold was exceeded (García-Caparrós et al., 2020), light exposure would not promote seedling growth and may even inhibit seedling development (Hwang et al., 2020; Song et al., 2022). The prediction model established in this study showed that the light recipe regulation patterns and the effected of light on seedlings were consistent.

### 4.2 Advantages of XGBoost in agricultural prediction

ZhengThe results showed that XGBoost is superior to other models in predicting seedling growth. When compared with MLR, Ridge Regression, RF, GRU, and LSTM, XGBoost demonstrated superior prediction timeliness and accuracy, thereby enhancing the model’s applicability in actual production scenarios. Leveraging its gradient boosting framework and tree ensemble structure, XGBoost excels at capturing the complex relationships between light recipes, hypocotyls, and seedling stems during nonlinear data processing. Simultaneously, regularization techniques embedded within the algorithm mitigate overfitting risks, thereby enhancing the model’s robustness and generalization capabilities. This was consistent with the results of Aasim (Aasim et al., 2022) and Yan (Yan et al., 2022). Especially in the real-time decision-making of the model, the potential and practicability were further emphasized (Ser and Bati, 2023). The research results of Wadhwa and Malik showed that XGBoost performs better than other models such as SVM in predicting agricultural results (Wadhwa and Malik, 2024). Tang also emphasized that XGBoost has become the most attractive means in the agricultural field because of its high precision and easy deployment (Tang et al., 2025). The feasibility of the tomato irrigation prediction model established by Wang based on XGBoost also confirms this point (Wang et al., 2025).

## 5 Conclusion

This study addresses the requirements of automatic grafting machines and proposes an evaluation method for tomato seedlings suitable for automatic grafting. The length of the hypocotyl and the diameter of seedling stem are key factors affecting the success rate and quality of automatic grafting. Using multiple lighting formulations during the growth cycle of seedlings can effectively alter the morphology and quality of the seedlings. A growth prediction model for tomato seedlings based on XGBoost was established, which showed high accuracy for both rootstock and scion predictions (R² = 0.9253 and 0.9334, respectively). Using the Bayesian optimization strategy, combined with Gaussian noise and eigenvalue perturbation, the hyperparameters of XGBoost were tuned. The results showed that the optimized forward prediction model was more accurate. The R² of the rootstock hypocotyl prediction model improved by 0.76%, the seedling stem prediction model improved by 0.43%, the scion hypocotyl prediction model improved by 0.87%, and the seedling stem prediction model improved by 0.56%. Use an Intelligent light control system based on a Raspberry Pi to cultivate rootstocks and scions, and verify the results through grafting experiments. The results indicated that the intelligent light control system can achieve targeted and precise cultivation of seedlings, and the seedlings cultivated using this model were more suitable for automatic grafting than commercially available seedlings, the automatic grafting success rate and post-grafting survival rate of light-regulated seedlings were 8.3% and 1.4% higher than those of commercially available seedlings, respectively. This demonstrates the feasibility of the model and highlights the practical application of the system in precision agriculture.

However, this system still has some shortcomings. Although this model has demonstrated good performance in prediction and regulation, its applicability may need to be adjusted according to different varieties of tomatoes. Furthermore, the growth of seedlings is influenced by multiple factors such as temperature, humidity, and wind speed, and this aspect merits further exploration. In the future, the accuracy of the model can be enhanced by expanding the dataset and obtaining the morphological parameters of the seedlings at multiple time points on the same day. Furthermore, exploring and optimizing the camera algorithm to enhance shooting accuracy is also one of the ways to reduce errors.

## Data Availability

The raw data supporting the conclusions of this article will be made available by the authors, without undue reservation.
